# Let’s ask the patient – composition and validation of a questionnaire for patients’ feedback to medical students

**DOI:** 10.1186/s12909-021-02683-y

**Published:** 2021-05-10

**Authors:** Karin Björklund, Terese Stenfors, Gunnar H. Nilsson, Hassan Alinaghizadeh, Charlotte Leanderson

**Affiliations:** 1 Division of Family Medicine and Primary Care, Department of Neurobiology, Care Sciences and Society (NVS), Karolinska Institutet, Stockholm, Sweden; 2 Department of Learning, Informatics, Management and Ethics, Karolinska Institutet, Stockholm, Sweden; 3 Academic Primary Health Care Centre, Region Stockholm, Stockholm, Sweden

**Keywords:** Clinical practice, Composition of questionnaire, Questionnaire validation, Medical students, Communication, Patient-centered, Patient feedback, Survey, Work-based learning

## Abstract

**Background:**

Adequate communication and maintaining a patient-centered approach throughout patient encounters are important skills for medical students to develop. Feedback is often provided by clinical teachers. Patients are seldom asked to provide feedback to students that systematically addresses knowledge and skills regarding communication and patient-centeredness during an encounter. One way for patients to provide feedback to students is through a questionnaire; there is, however, a lack of such validated feedback questionnaires. This study aimed to compose and validate a feedback questionnaire for patients’ feedback to medical students regarding students’ ability to communicate and apply patient-centeredness in clinical practice.

**Method:**

This study comprises (a) composition of the questionnaire and (b) validation of the questionnaire. The composition included (1) literature review, (2) selection and composition of items and construction of an item pool, (3) test of items’ content, and (4) test of the applicability of the questionnaire. The items originated from the Calgary-Cambridge Guide (Kurtz S, Silverman J, Benson J and Draper J, Acad Med 78:802-809, 2003), the ‘Swedish National Patient Survey’ (National Patient Survey, Primary Health Care, 2020), patient evaluation form by Braend et al. (Tidsskr Nor Laegeforen 126:2122–5, 2006), and additional developed items. The items were further developed after feedback from 65 patients, 22 students, eight clinical supervisors, and six clinical teachers. The validation process included 246 patients who provided feedback to 80 students. Qualitative content analysis and psychometric methods were used and exploratory factor analysis assessed internal validity. Cronbach’s alpha was used to test the reliability of the items.

**Results:**

The process resulted in the 19-item ‘Patient Feedback in Clinical Practice’ (PFCP) questionnaire. Construct validity revealed two dimensions: *consultational approach* and *transfer of information.* Internal consistency was high. Thematic analysis resulted in three themes: *ability to capture the personal agenda of the consultation*, *alignment with the consultation*, and *constructs and characteristics.* Students reported that the PFCP questionnaire provided useful feedback that could facilitate their learning in clinical practice.

**Conclusions:**

The results of this study indicate that the questionnaire is a valid, reliable, and internally consistent instrument for patients’ feedback to medical students. The participants found the questionnaire to be useful for the provision of feedback in clinical practice. However, further studies are required regarding the PFCP questionnaire applicability as a feedback tool in workplace learning.

## Background

Patient-centeredness is considered a key component to achieve high-quality care and increase patients’ participation in their own healthcare [1–4]. A patient-centered approach and related working methods include a framework for dialogue [5], a transfer of knowledge, patient and physician autonomy, and consultation skills [6]. The conceptualisation of *patient-centered care* has been developed over time, and various aspects and dimensions of the consultation have been enhanced [7–10]. Frameworks for patient-centered care have served as bases for evaluating various patient perspectives and experiences of healthcare [11]. Research has shown that patients would like to be more involved during the patient encounter [11], and that a focus on patients’ experience and knowledge during the encounter can contribute to more patient-centered care [12]. In 2015, a new version of the *Swedish Patient Act* presented to further strengthen patients’ role as collaborators in their own care [1]. Despite quality improvements and educational interventions, measurements and reports have repeatedly identified areas for improvement in patient care, including in dimensions such as consent, participation, and information [2, 3].

Research and reports have highlighted communication methodologies as tools for improving dialogue, patients’ satisfaction, and participation in their own care [13]. Communication and clinical skills are important core competencies for medical students to practice and develop [13–15]. Undergraduate medical education often involves early training in clinical skills and communication. Despite students’ education and training in communication and patient-centeredness, research has shown that students’ attitudes often shift from being patient-centered early in education to later being represented by a more traditional doctor-centered paternalistic approach [16, 17]. However, through specific and continuous education, students can develop and maintain an ability to apply a patient-centered approach throughout their education [17]. Students’ clinical supervisors are often the main providers of feedback regarding communication and patient-centeredness [18]. Patients’ participation in medical education can facilitate students’ abilities to evaluate their own communication and patient-centeredness proficiencies, positively influencing patients’ experiences of healthcare [19, 20]. Traditionally, patients’ involvement in medical education has been passive and objectified, hence illustrating specific conditions or clinical findings [20]. Over the last decades, patients’ involvement in various levels of medical education has evolved [21, 22]. However, patients seldom participate on a regular basis in medical students’ learning by providing feedback that directly targets knowledge and skills regarding communication and patient-centeredness from the patient’s perspective during a patient encounter [19, 23]. Research has shown that medical students often experience patients’ written feedback as generally encouraging, moderate, and positive [19] and more seldom as a substantial source of information that identifies students’ knowledge levels and knowledge gaps [24, 25].

Patient questionnaires provide an opportunity for patients to provide feedback in medical education. Internationally, a plethora of questionnaires have been developed for patients’ feedback to healthcare providers and medical doctors during residency [14, 26–28]. However, only a few questionnaires have been developed for patients’ feedback to medical students [25, 29, 30]. Previous questionnaires often target patients’ delayed, anonymous, nonspecific, and global feedback regarding experiences of provided healthcare. Moreover, these questionnaires have often included questions that asked patients to rate a student’s overall performance in certain domains, rather than concrete questions about patients’ subjective experience of the respective part of the consultation. For example, a currently available questionnaire may have a question such as, *‘Was the student telling you what you wanted to know about your symptoms and/or illness?’* [31], rather than asking the patient, *‘Did the student provide information about your symptoms and/or illness?’,* and *‘Did the students provide information about your symptoms and/or illness in a way you understood?’* in order to make the feedback more actionable for the student. Patients have also often been asked to perform a global assessment of students as future healthcare workers through questionnaire items such as, *‘Imagine for a second that you could change to a new dedicated family physician; would you consider changing to he/she (the student) as your physician?’* [29]. However, while this type of feedback can be gratifying for students to receive, it is not actionable as a learning tool [32]. Furthermore, the content and structure of existing questionnaires are not in direct alignment with learning goals regarding patient-centeredness in medical education [7, 10].

The aim of this study was to compose and validate a feedback questionnaire for patients’ feedback to medical students regarding students’ ability to communicate and apply patient-centeredness in clinical practice.

## Methods

### Settings

This study was conducted between March 2016 and May 2018 at primary health care (PHC) centres in Stockholm County. To address the study’s aim, this research comprised (a) composition of the questionnaire and (b) validation of the questionnaire.

### Context

Within the medical programme, Karolinska Institutet (KI), Stockholm Sweden, students perform clinical rotations at PHC centres during semesters one to11 (excluding semesters eight and 10). These placements last between four to 7 days per semester. Students’ early clinical training focuses on patients’ agendas and clinical examination [10]. Clinical reasoning during students’ early training is often addressed in dialogue between supervisors and students. The information that the students receive through the patients’ agendas is incorporated into the supervisor’s clinical reasoning and mutual agreement in order to highlight the importance of patient-centeredness as an entity throughout the patient encounter early in the students’ learning. In collaboration with their clinical supervisor, starting in semester three, students initiate training in clinical reasoning. Starting in semesters five and six, under supervision, students initiate training in performing the mutual agreement with patients. During semesters nine to 11, students perform the entire patient encounter under supervision.

### Participants

The participants in this study were medical students from semesters two, four to seven, nine, and 11 who performed clinical practice at PHC centres. The study inclusion criteria were patients at the PHC centres, aged 18 years and older, and the exclusion criteria were patients with dementia, cognitive disabilities, or mental disorders.

Participants were invited into this study by e-mail (heads of PHC centres, medical students, and clinical supervisors). Patients were invited to participate at PHC centres through oral and written information and consent before the encounter at the PHC.

### Composition of the questionnaire

**Fig. 1:**
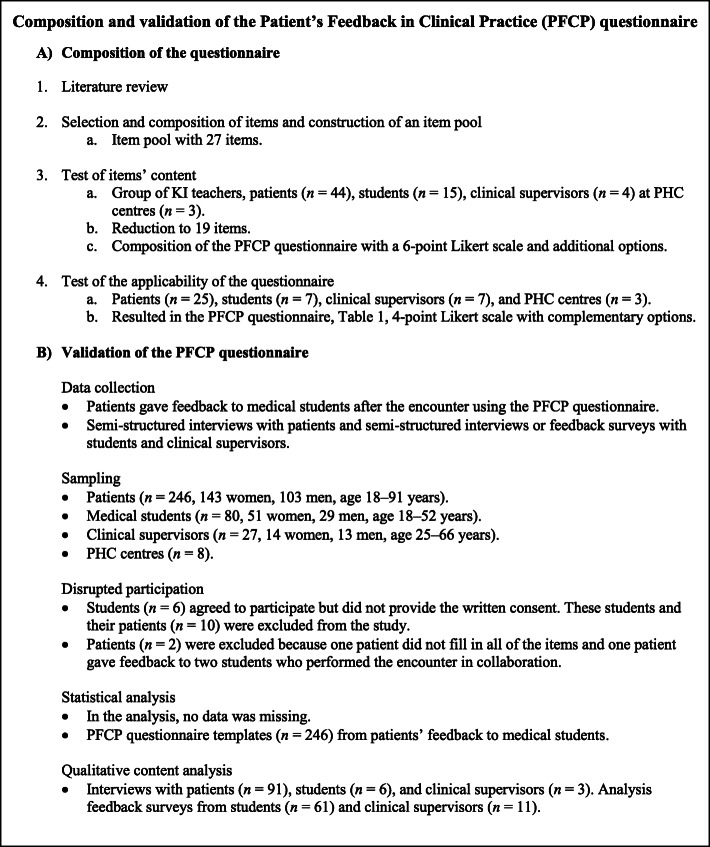
Flowchart of the (**A**) composition and (**B**) validation of the PFCP questionnaire

The composition of the questionnaire included: (1) a literature review, (2) selection and composition of items and construction of an item pool, (3) test of items’ content, and (4) test of the applicability of the questionnaire (Fig. 1) [33, 34]. The ‘Consensus-Based Standards for the Selection of Health Measurement Instruments’ (COSMIN checklist) [35] was used as a guide in the composition process.

#### Literature review

A literature review was undertaken to identify existing questionnaires designed for patient feedback to medical students, residents, and specialists, with a focus on communication and patient-centeredness [36]. Before the literature review, key concepts were defined in order to target current models for communication and patient-centeredness that were in alignment with students’ learning goals.

Key concepts for the literature review were defined using:

- the ‘Swedish National Patient Survey’ (information, knowledge, involvement, participation, respect, and attitude) [26]
- the National Board of Health and Welfare guidelines for person-centered care [12]
- Calgary-Cambridge guide [7]
- the Pendleton model [9]
- the generic model of doctor-patient communication developed at Maastricht Medical School [10]
- the learning goals for Swedish medical education at KI regarding communication and patient-centeredness, and models of learning and training in workplace-based education [37]

A literature search was performed on PubMed, Web of Science, and Google Scholar. The following MeSH (medical subject headings) terms and key concepts were used: **‘**medical education’, ‘assessment’, ‘patient feedback’, ‘patient satisfaction’, ‘communication skills’, ‘questionnaire’, ‘patient-centeredness, ‘clinical competence’, ‘medical student’, ‘student learning’, and ‘family practice’.

Based on the literature review, inclusion and exclusion criteria for patient feedback questionnaires and aspects of patient-centeredness and communication were determined (Table 1).

**Table 1:** Inclusion and exclusion criteria for patient feedback questionnaires

Inclusion	Exclusion
Designed for patient feedback to medical students, or residents, or specialists	Designed for multi-source, peer, or observer feedback
Provide individual performance feedback	Not used for individual feedback
Assesses key content regarding communication and patient-centeredness skills	Does not assess key content regarding communication and patient-centeredness skills
Dimensions in alignment with learning goals and national patient survey	Items focus on technicality and organisation, e.g. practice setting and administration

The literature review resulted in 841 articles identifying 68 questionnaires for patient feedback based on inclusion and exclusion criteria. Three of the questionnaires were intended for patients’ feedback to medical students [25, 29, 30], and 65 of the questionnaires were identified as a form for provision of patient feedback to specific clinics, doctors, and residents, of which 12 questionnaires had been designed for educational purposes. None of the identified questionnaires included items that were all in alignment with the inclusion criteria of the current study.

#### Selection and composition of items and construction of an item pool

Items in the 68 questionnaires in alignment with the study inclusion criteria, with content relevant to Swedish medical care and education in the dimensions of information, knowledge, involvement and participation [25], served as the basis for the selection process. All questionnaires included several items with similar content in alignment with the study inclusion criteria, e.g. *‘Were you involved as much as you wanted to be in decisions about your care and treatment?’* [26, 38]. All questionnaires also included items that provided feedback exploring both patients’ satisfaction with and experience of a consultation. Items that measured more than one aspect, items that provided non-concrete feedback (e.g. judgmental adjectives), and items that included verbs describing emotions (e.g. *‘Do you feel this doctor listened to you?’*) [38], were excluded in the selection process. The Swedish national patient survey and learning objectives at the KI medical programme served as important guideline documents in order to enable possible comparisons in future studies. The selection process resulted in 41 questionnaires for item content analysis by two of the study authors (K.B. and C.L.). A subsequent reduction process yielded seven questionnaires, which were documented in a spreadsheet that contained all seven questionnaire items. This list was reviewed by an expert group comprising four clinical lecturers at the Division of Family Medicine and Primary Care, KI. These experts were responsible for teaching patient-centered communication techniques at the medical programme, KI, as well as in residency and CME courses at a national level. These experts read the list repeatedly before selecting items that they perceived best to correlate to a certain domain in the intended learning outcomes regarding communication and patient-centeredness. The result was then processed repeatedly by the team of authors, and the final selection of items was confirmed by the expert group, reaching a consensus.

In total, 27 items were selected from the a) ‘Swedish National Patient Survey’ (*n* = 12) [26], b) the Calgary-Cambridge Guide (*n* = 11) [7], and c) patient evaluation form by Bread et al. (*n* = 1) [29]. Three complementary items were developed in order to include aspects of patients’ perceived experience of consultation and participation information throughout their patient encounter, according to inclusion criteria – for example, ‘*Did the student ask if the information you were given was interpretable?’* [34]. Considerations of items were made with the intention to reduce the ceiling effect of the patients’ assessments. The selected items were reframed and modified – for example, in the direction towards a subjective perception of the patient encounter – and worded as open-ended, direct questions (e.g. *‘Did you have the opportunity to tell the student in your own words about your problem?’* was changed to *‘Did you have the opportunity to explain, in your own words, the reason for your visit, or what has happened since you last visited the doctor?’*).

#### Test of items’ content

To determine how well the 27 items’ content captured the intended aspects of patient-centered communication (face validity) and allowed patients to provide, and students to receive, patients’ feedback about the patient encounter, discussions were held with a group of four content-experts who participated in the selection process. The content-experts are clinical lecturers at the Division of Family Medicine and Primary Care, KI. At three PHC centres, semi-structured interviews were conducted with patients (*n* = 44) before or after a patient encounter. An initial evaluation of the result of patient interviews performed before consultation showed that some items were often interpreted as similar. However, after the patients had experienced their consultation, they no longer considered the items similar and believed that the items would provide valuable differential feedback on the subjects in the items. The items were evaluated for their ability to target important areas of feedback, clarification of feedback subject, comfort in providing feedback in the respective item content area, and linguistic interpretability. The items were also evaluated by students (*n* = 15) and clinical supervisors (*n* = 4) through semi-structured interviews during students’ clinical placement at PHC centres [34]. These interviews were transcribed and analysed using deductive content analysis [39], which provided information for the selection and inclusion of items to the questionnaire. Throughout this process, items were modified and reduced to minimise overlap and to direct patients’ attention in the items towards patient-focus, rather than student-focus during the encounter, e.g. ‘*Did the student ask if there was something that you were worried about regarding your problem?’* was changed to *‘Did you have the opportunity to explain if there were something that worried you regarding your problem?’*

The item composition process resulted in 19 items (items 1–8, and 14 were derived from the Calgary-Cambridge Guide [7]; items 10–12, 15–16, and 18–19, were derived from the ‘Swedish National Patient Survey’ [26]; items 1, 3, and 14 occurred both in the Calgary-Cambridge Guide and the ‘Swedish National Patient Survey’; item 17 was derived from the patient evaluation form by Braend et al.17 [29], and items 9 and 13 were formulated in discussions between the research group and experts. The items were connected to a six-point Likert scale with clarifying text for each scale step (from *strongly disagree* to *strongly agree*). *‘Not applicable’* and *‘Performed by supervisor’* was included as an additional option, and space for *free-text feedback* was included at the end of the questionnaire.

#### Test of the applicability of the questionnaire

##### Data collection tools: written surveys and an interview guide to evaluate the questionnaire during the applicability test

Before the PFCP questionnaire applicability test, two evaluation surveys were formulated to explore students’ and clinical supervisors’ experiences of the PFCP questionnaire [40]. The evaluation surveys were also used as a guide for semi-structured interviews with students and clinical supervisors during the applicability test of the questionnaire.

##### Data collection during the applicability test of the questionnaire

To test the applicability of the PFCP questionnaire for provision of feedback and usefulness as a learning and teaching tool, patients (*n* = 25) completed the questionnaire with feedback to medical students (*n* = 7) directly after the encounter in PHC centres (*n* = 3). After the patients had completed the PFCP questionnaire, they were interviewed by the use of a semi-structured interview guide, to explore the questionnaire’s ability to capture their perspectives and experiences during an encounter. Students and clinical supervisors (*n* = 7) completed an evaluation survey or were interviewed to explore the perceived usability of the patient feedback from the PFCP questionnaire as a tool for learning and clinical supervision. K.B. (first author) collected these PFCP questionnaire forms and evaluation surveys for analysis.

Data from the PFCP questionnaire regarding the patients’ experience during the student-led encounter was analysed; results are not presented in this paper. Data from the evaluation surveys and interviews were analysed using inductive qualitative content analysis [39]. The questionnaire applicability test showed that the clinical supervisors tended to disregard the patients’ use of the entire six-point Likert scale in rating students’ perceived performance. Instead, the clinical supervisors often interpreted the patients’ ratings in the 5–6 range as indicating an overall adequate student performance, disregarding the patients’ intent to suggest areas for students’ improvement. The Likert scale was therefore changed from a six-point Likert scale to a four-point Likert scale in order to address this concern and reduce the observed ceiling effect. To further provide students with interpretable and useful feedback, space was included for free-text comments after each item (Table 2).

**Table 2:** The 19 items included in the Patient’s Feedback in Clinical Practice (PFCP) questionnaire^a^

1.	Did you have the opportunity to explain the reason for your visit or what had happened since you last visited the doctor? (F1)
2.	Did you have the opportunity to explain your own thoughts regarding your problems? (F1)
3.	Did you have the opportunity to explain if there was something that worried you regarding your problems? (F1)
4.	Did you have the opportunity to express if there was something specific you wanted to be performed/initiated during the consultation? (F1)
5.	Did the student confirm with you that he/she understood your cause of concerns correctly by summarising what you told him/her? (F1)
6.	Did the student explain his/her medical questions, so you understood why they were asked? (F2)
7.	During the clinical examination, did the student explain why certain examinations were performed? (F1)
8.	Did the student take into consideration your own thoughts regarding your problem when you discussed the follow-up plan/treatment? (F2)
9.	Did you receive information/explanation from the student which made it possible for you to participate in the planning of care/treatment? (F2)
10.	Did the student provide information about suggested care/treatment in a way that you understood? (F2)
11.	Did the student provide information about medication in a way that you understood? (F2)
12.	Did the student provide information in a way that you understood regarding symptoms that call for immediate contact with healthcare? (F2)
13.	Did the student ask if the information you were given was interpretable? (F2)
14.	Did you have the opportunity to bring up questions you had before the visit regarding your cause of concerns? (F2)
15.	Did the student involve you in the decision-making process regarding your care/treatment? (F2)
16.	Were you involved in the decision-making process regarding your care/treatment to the extent you wanted? (F2)
17.	Are you satisfied with the initial plan that was decided upon together with the student? (F2)
18.	Did you experience that the student treated you with compassion and consideration? (F1)
19.	Did you experience that the student treated you with respect and dignity? (F1)

^a^Each item is marked with Factor 1 (F1) or Factor 2 (F2) to illustrate the division (result from the validation of the questionnaire) of the items into two dimensions: the consultational approach (F1) and the transfer of information (F2)

### Validation of the questionnaire

#### Data collection during the validation of the questionnaire

Using the PFCP questionnaire, patients provided feedback to medical students. The feedback was provided after an encounter performed by the medical student, under the supervision of a clinical supervisor. Semi-structured interviews were conducted with patients to evaluate their experiences with the PFCP questionnaire. The student and the clinical supervisor received the patient feedback at the end of the same day on which the encounter with the patient had taken place and the patient feedback provided. After having taken into account the patient feedback, students and clinical supervisors completed an evaluation survey or were interviewed to evaluate the PFCP questionnaire as a tool for medical students’ clinical education. The evaluation surveys and interview guide from the applicability test were also used to validate the questionnaire. K.B. collected the PFCP questionnaire and evaluation surveys for analysis.

#### Participants and sampling

In total, 246 patients (143 women and 103 men, ages 18–91), 80 medical students (51 women and 29 men, ages 18–52), and 27 clinical supervisors (14 women and 13 men, ages 25–66) at PHC centres (*n* = 8) in Stockholm City County participated in the validation of the questionnaire. Six additional students agreed to participate but did not provide written consent; these students and their patients (*n* = 10) were excluded from the study. Two additional patients were excluded, one of whom did not completely fill in all of the questionnaire items and one of whom provided feedback to two students who had collaborated during the patient encounter.

#### Analysis

##### Statistical analysis: internal consistency, construct validity, and reliability

Exploratory factor analysis (EFA) was used to assess how well the items of the PFCP questionnaire measured what they were intended to measure (content validity) and to explore associations among the items (internal validity). Furthermore, EFA was used to control any grouping tendency between the items, discern underlying factors within each factor, and reduce items that might be distributed across more than one factor [34]. Oblique rotation was used to clarify the items’ grouping [34]. After changing the Likert scale in the questionnaire to a four-point Likert scale, the ceiling effect that had been noted during the test of the applicability of the questionnaire was found to be less prominent. Related confounders (patient age and gender; student age, gender, and current semester) of these items were controlled by multivariate analysis of covariance (MANCOVA) (results not shown in this paper). Testing of the items’ magnitude in the factor models and internal consistency of the item construct was controlled by Cronbach’s alpha coefficient [34]. Cronbach’s alpha values from 0.6 to 1.0 were considered acceptable [34]. SAS 9.4 (SAS Institute Inc., Cary, NC) software was used for the statistical analyses.

##### Qualitative content analysis: participants’ experiences in the validation of the questionnaire

Data from the transcribed semi-structured interviews with patients (*n* = 91), students (*n* = 6), and clinical supervisors (*n* = 3), and text from the written evaluation surveys from students (*n* = 61) and clinical supervisors (*n* = 11), was analysed using qualitative content analysis [39].

The qualitative data were analysed using inductive qualitative content analysis.

- The interviews were audiotaped and transcribed, and the students’ and clinical supervisors’ written feedback from the evaluation surveys was documented.

Interviews

- The text from the transcribed interviews was read repeatedly for a global understanding by K.B. and C.L., and notes were taken.
- Meaning units were identified.
- The units were condensed according to perceived key content areas.
- The units were compared in order to ensure consistency.
- The meaning units were sorted into categories established by K.B. and C.L.

Evaluation surveys

- The text from the evaluation surveys was read repeatedly for global understanding by K.B. and C.L., and notes were taken.
- The units were condensed according to perceived key content areas.
- The units were compared in order to ensure consistency.
- The meaning units were sorted into categories established by K.B. and C.L.

Final step

- The underlying meanings of the categories from the interviews and evaluation surveys were interpreted and merged, resulting in three themes which were established by K.B. and C.L.
- These three themes are presented in the ‘Results’ section below.

Quantitative data from the evaluation survey’s mean and range were documented for each question (presented under respective themes in the ‘Results’ section below).

## Results

### Statistical results: internal consistency, construct validity, and reliability

The exploratory factor analysis resulted in two dimensions: consultational approach (F1) and transfer of information (F2). F1 includes items 1–5, 7, and 18–19, and F2 includes items 6 and 8–17. The two dimensions’ values from EFA and revised factor analysis [41] are presented in Table 3, whose rows correspond to the variables from items 1–19 and whose columns correspond to F1 and F2, with variance explained by each factor before and after rotation, mean (SD), and Cronbach’s alpha (when items were removed). The internal consistency of the scale was interpreted as high. The Cronbach’s alpha coefficients ranged between 0.89 and 0.91. No data was missing in the analysis process.

**Table 3:** Factor loading and descriptive analysis of the PFCP questionnaire^a^

	Factor loads EFA		Mean (SD)	Cronbach’s alpha (when items are removed)
	F1	F2		
Item - 1	−0.07	**0.84**	4 (0.70)	0.91
Item - 2	−0.05	**0.84**	4 (0.66)	0.90
Item - 3	0.01	**0.80**	4 (0.95)	0.90
Item - 4	0.10	**0.66**	4 (1.28)	0.90
Item - 5	0.20	**0.69**	4 (0.86)	0.90
Item - 6	**0.56**	0.42	4 (1.38)	0.89
Item - 7	0.31	**0.51**	4 (1.33)	0.90
Item - 8	**0.70**	0.40	4 (1.48)	0.89
Item - 9	**0.89**	0.10	4 (1.55)	0.89
Item - 10	**0.92**	−0.03	4 (1.70)	0.90
Item - 11	**0.73**	0.04	2 (1.96)	0.90
Item - 12	**0.71**	0.07	3 (1.88)	0.90
Item - 13	**0.80**	0.13	4 (1.52)	0.90
Item - 14	**0.77**	0.22	4 (1.43)	0.89
Item - 15	**0.82**	0.19	4 (1.61)	0.89
Item - 16	**0.80**	0.23	4 (1.51)	0.89
Item - 17	**0.86**	0.08	3 (1.44)	0.89
Item - 18	0.26	**0.61**	4 (0.44)	0.90
Item - 19	0.33	**0.59**	4 (0.29)	0.90
Variance explained by each factor before and after rotation	10.45 9.72	2.28 6.59		

^a^The exploratory factor analysis (EFA) for factor 1 (F1) and factor 2 (F2) with the variance explained by each factor before and after rotation. Mean (SD) for each item and Cronbach’s alpha for each item (when items are removed)

### Qualitative results: participants’ experience of validation of the questionnaire

The thematic analysis of data from the validation of the questionnaire, including the evaluation survey and interviews, resulted in three themes: *ability to capture the personal agenda of the consultation*, *alignment with the consultation*, and *structure and content.* Table 4 shows a summary of patients’, students’, and clinical supervisors’ perspectives of the PFCP questionnaire as a pedagogical feedback tool.

**Table 4:** Summary of patients, students, and clinical supervisors’ perspectives of the PFCP questionnaire as a pedagogical feedback tool

Identified themes	Patients’ perspectives	Students’ perspectives	Clinical supervisors’ perspectives
**Ability to capture the personal agenda of the consultation**	Clarifies the provided information and suggested treatment in relation to personal agenda	Identifies students’ assignment as health providers regarding communication and consultation skills	Identifies supervisors’ pedagogical assignment
**Alignment with the consultation**	Visualises the consultation process with preserved authenticity	Visualises the expected performance in relation to learning goals	Highlights and illustrates learning outcomes and enhances the framework for feedback in alignment with the consultation
**Construct and characteristics**	Visualises the intended task as a feedback provider. Facilitates the process to provide specific feedback	Facilitates the identification of the level of knowledge and areas for improvement	Facilitates the clinical supervisor’s ability to provide adequate feedback to students

#### Theme 1: Ability to capture the personal agenda of the consultation

##### Patients

The questionnaire provided the patients with a tool that facilitated their interpretation of the consultation in relation to the patient’s personal agenda, e.g. questions that had been asked, examinations that had been performed, information that had been provided, and decisions that had been mutually agreed upon. The questionnaire was also perceived to clarify important aspects of the patient’s own care.

*‘I thought it was good, very straightforward, and so, very easy to separate the parts. I thought it described the visit pretty well, what we went through and so.*. *.’*

##### Students

Students (*n* = 61) evaluation surveys explored how patients’ feedback helped them visualise the pedagogical assignment a student has during a patient encounter (using a 4-point Likert scale, mean 3.5).

The students stated that an awareness of their assignment in relation to the patient was further clarified. The medical assignment, to recognise and interpret symptoms and perform an adequate clinical examination, the students stated the necessity to provide patients with clarifying information throughout the entire consultation. The pedagogical assignment to facilitate the process of mutual agreement, by considering the patient’s level of knowledge and concerns, was perceived as targeted through the patient’s feedback.

*‘I received a greater understanding, that the clinical examination is not only for me to find a diagnosis, but also for the patient to feel well examined’.*

*‘It made me realise. .. next time I should explain a little more about what I examine etc.’.*

##### Clinical supervisors

The patients’ perspectives obtained from the PFCP questionnaire were believed to underpin the clinical supervisors’ pedagogical assignment to provide feedback regarding the students’ level of patient-centeredness. The PFCP questionnaire was also considered to facilitate and legitimise dialogue with students regarding important aspects of patient-centeredness within each part of a consultation.

*‘It is important to think about all the steps, such as medicines and further diagnostic interventions. It is easy to forget certain steps during the feedback, the patient’s feedback strengthened and provided a structure for the feedback. .. ’.*

#### Theme 2: Alignment with the consultation

##### Patients

The authenticity of the questionnaire regarding the structure and content of the consultation was perceived by the patients to be high.

‘*They [the items] were in alignment with the experienced encounter; it almost felt as if he was asking questions directly from the questionnaire’.*

##### Students

Students believed the questionnaire to concretise and target learning goals and to provide structured feedback throughout the consultation.

*‘..*. *highlighted all the parts that should be included in the patient consultation’.*

*‘The importance of the summary to provide clarity. .. ’.*

Students experienced that patients’ feedback highlighted the importance of including a patient-centered approach in dialogue with patients in order to increase patients’ participation during the encounter.

*‘The importance of. .. responding to the patient’s questions [and] taking the patient’s agenda into consideration. .. became very clear in the feedback. .’.*

##### Clinical supervisors

The questions in the PFCP questionnaire were found to be in alignment with the expected structure and content of a patient encounter. The questions were also perceived to facilitate the supervisors in the identification of the necessity to provide feedback to students regarding patient-centeredness.

*‘The questionnaire is designed in alignment with the consultation’.*

*‘... I have not previously focused enough on feedback during the final parts of the patient encounter, such as how the patient has perceived the encounter and, for example, how the student has ensured that the patient understands … ’.*

#### Theme 3: Construct and characteristics

##### Patients

The patients experienced that the questionnaire targeted important content and strengthened their ability to provide relevant feedback to the students.

*‘... the items were good, they adequately visualised what the student could do and what she did’.*

*‘It was good, it was both the human factors about how to talk to the patient as well as the medical, so it was both’.*

The questionnaire allowed the patients to state which parts of the consultation were performed by the student and the clinical supervisor, respectively. However, in some cases, patients found it challenging to isolate a student’s performance from the interference of the clinical supervisor.

*‘Was it the student who did it well, or was it this doctor who did it well? It is good that I can also mark that the supervisor performed’.*

Patients described that the items clarified their experience of the encounter and facilitated their provision of feedback. However, patients also regarded the opportunity to write free-text comments as important.

*‘That I had the opportunity to write my own answers was really good’.*

##### Students

The students’ (*n* = 61) evaluation survey indicated that patients’ feedback provided valuable information regarding students’ abilities to apply patient-centered communication (3.4 out of 4 on the Likert scale), as well as guidance for continuous training in clinical skills (3.2 out of 4 on the Likert scale).

*‘Clear feedback when a patient experienced that I answered their ideas, concerns and expectations’.*

##### Clinical supervisors

The clinical supervisors’ (*n* = 22) evaluation survey indicated that patients’ feedback could provide valuable information regarding students’ abilities to apply patient-centered communication (3.3 out of 4 on the Likert scale), as well as guidance on students’ continued training in clinical competencies (2.7 out of 4 on the Likert scale), while also facilitating the students’ visualisation of their pedagogical assignment in their dialogue with patients (3.3 out 4 on the Likert scale).

Clinical supervisors stated that patients’ feedback added perspective and provided valuable, applicable information for their own feedback to students regarding how to communicate and provide information to patients.

*‘The questions were so specific that I could give concrete examples of how to do things differently’.*

*‘You received more information about how the student responds to the patient and solves problems’.*

Overall, clinical supervisors expressed that the time invested in using the PFCP questionnaire was proven to be well spent. Some clinical supervisors declined to participate due to stress and time loss incurred. However, very few clinical supervisors maintained such hesitation after participating in this study.

*‘Structured, good, but sometimes time-consuming’.*

*‘Literally complicated at first. Took a little longer but provided a very good structure’.*

## Discussion

This study focused on the composition and validation of a feedback questionnaire to allow patients to assess their experiences of core communication and patient-centeredness aspects during a patient encounter in order to provide medical students with feedback for the identification of knowledge gaps and areas for development. The composition of the items resulted in a questionnaire with 19 items (nine partly adapted items from the Calgary-Cambridge Guide [7], seven items from the ‘Swedish National Patient Survey’ [26], one item from the patient evaluation form by Braend et al.17 [29], and two complementary items). The results from analysis and interpretation of data indicated that the PFCP questionnaire is a valid, reliable, and internally consistent instrument for patients’ feedback to medical students.

The item selection process followed a reductive and adaptive process, including mixed methods, to support content and face validity. In the selection process a framework for communication and patient-centeredness (Calgary Cambridge Guide [7] and Maastricht Medical School [10]) was used and included an initial evaluation with experts and evaluations with patients, students, and clinical supervisors during several steps [34, 35].

To consider whether power in the psychometric evaluation was adequate, the most crucial consideration is the relationship between how well the items’ loaded on factors and the study’s sample size. The recommended sample size is approximately 10 participants for each item. Taking these factors into account, the sample size of 246 was considered reliable [42].

To provide evidence for construct validity, reliability, and internal consistency, several psychometric assessments were performed in alignment with previous studies [43]*.* Cronbach’s alpha supported the inclusion of each item in the questionnaire and showed that none of the items measured the same construct, which supported both the reliability of the two-factor structure and the internal consistency [34]. Background factors did not affect the factor structure significantly [33], even though the distribution was disproportionate concerning gender, age, and study years, which also supported the items’ inclusion in the questionnaire.

Analysis of the interviews from the validation process revealed coherent data that did not suggest further aspects compared to the data obtained from the written evaluation surveys. The process for analysing data from the evaluation surveys and interviews was identical and clearly described to detect dependability issues.

Statistical [33, 34] and content analysis [39] confirmed that the two factors sufficiently covered the core aspects of communication and patient-centeredness, in alignment with other studies [7, 10, 26]. The items’ content enables patients to provide concrete, interpretable, and actionable feedback regarding their experiences during an encounter, as previous studies have described [44].

During the composition process, the Likert scale was changed from a six-point to a four-point scale. The intention guiding this change was that students and clinical supervisors should be able to discriminate between and interpret the patients’ feedback to identify areas for improvement. Students and clinical supervisors described that the feedback identified important areas for improvement in alignment with students’ learning goals regarding communication and patient-centeredness [37]. The alteration of the scale, by using fewer scale steps, indicated that the four-grade Likert scale more clearly identified an area for improvement than the six-grade Likert scale.

Patients provided high-score feedback for the items in the dimension *consultational approach*, which aligned with the results of previous studies [30, 45]. For the second dimension, *transfer of information*, the ceiling effect observed was slightly lower, which is also in alignment with previous studies [29, 30, 45], and suggested that students, in general, are skilful at applying patient-centered communication in certain areas through their training, for example, in history taking [29].

In some previous studies, students had not expressed an interest in receiving further feedback from patients [29, 30]. However, in the current study, students regarded patients’ feedback as valuable for inclusion in their self-regulated learning process. This finding could perhaps be explained by the structure and content of the questionnaire. To further provide concrete feedback, patients were able to add written free-text clarifying comments. Both in this study and previous studies, patients considered the opportunity to clarify their feedback to be important [29, 46]. Several patients in this study also reported that the items adequately encapsulated their feedback regarding their experience of the encounter, stating that further clarification, therefore, was unnecessary.

The authors of previous studies and patient surveys have often advocated for anonymous patient feedback as a favourable approach to creating a safe environment in which to provide feedback regarding patients’ subjective experiences of an encounter [12, 30]. In the current study, patients were not anonymous, which could have affected their willingness both to participate in the study and provide feedback due to their plausible dependability upon their caregiver [19, 47]. The patients in this study indicated that the items’ content gave them opportunities to respond to specific aspects of an encounter and to assess concrete, non-emotional aspects of their experience. Students also confirmed that they had perceived the questionnaire items as concrete, interpretable, and actionable, visualising important aspects to develop regarding patient-centered communication techniques. Students also reported that their ability to relate patients’ feedback to their own experience of a particular encounter was important in order to facilitate their identification of learning gaps to address in further clinical training.

The clinical supervisors who participated in this study suggested that the PFPC questionnaire had clarified their pedagogical assignment to provide feedback regarding patient-centered communication. Furthermore, they reported that the questionnaire provided a structure for providing feedback, which to our knowledge, has not been discussed in previous studies [29, 30, 48]; however, these aspects should be explored further.

### Strengths and limitations

One strength of this study is that the composition and validation of the PFCP questionnaire used mixed methods and extensive data, which were collected and analysed using both statistical and qualitative methods in an effort to compose a valid, reliable questionnaire for patient feedback. The items originating from previously described and established questionnaires further added face validity to the PFCP questionnaire. A second strength is that patients, students, and clinical supervisors all reported that the PFCP questionnaire had targeted important clinical competencies in the areas of communication and patient-centeredness.

The PFCP questionnaire was composed in a Swedish medical education context, which could be considered a limitation. However, the content was based on common theories of communication and patient-centeredness, with offspring from work in alignment with the Calgary Cambridge Guide [7] and Maastricht Medical School [10], communicational guides commonly used in Western medical education. A second limitation could be that patients might find it difficult to discriminate whether the student or the clinical supervisor was the actual main provider of the given information and take-home message. Neither students nor clinical supervisors commented on these elements in their reflections on the questionnaire’s feedback applicability. A third limitation could be that data from the test of the items’ content were analysed using deductive content analysis. However, during this part of the process, the intention was to evaluate selected items related to the theories and knowledge of the subject communication and patient-centredness [49]. All other data was analysed using inductive content analysis to explore the participants’ perspectives and experiences of the PFCP questionnaire as a feedback provider [49].

### Implications for medical education and future research

Our results indicate that the PFCP questionnaire could serve as a valuable tool for increasing patients’ participation in medical students’ workplace learning. However, further analyses are required to explore students’ learning as a result of the PFCP questionnaire, assessed in a summative setting. Patients’ feedback obtained through the PFCP questionnaire could perhaps also serve as a progressive indicator of the level of knowledge and clinical competences in relation to milestones throughout medical education. Exploring how clinical supervisors can use feedback from the PFCP questionnaire as an addition to their own feedback requires further research. The fact that patients experienced a clarification of the encounter’s structure and content while completing the PFCP questionnaire could be a subject for further studies exploring how to provide patients with knowledge about patient-centered working methods.

## Conclusions

The results of this study indicate that the questionnaire is a valid, reliable, and internally consistent instrument for patients’ feedback to medical students. Patients, students, and clinical supervisors found the PFCP questionnaire provided useful feedback that could facilitate students’ learning regarding communication skills and patient-centeredness in clinical practice.

## Data Availability

The data generated and analysed during the current study are not publicly available due to ethics approval.

## Abbreviations

COSMIN checklist: Consensus-based Standards for the selection of health status Measurement Instruments
EFA: Explorative factor analysis
F1: Factor 1
F2: Factor 2
KI: Karolinska Institutet
MANCOVA: Multivariate analysis of covariance
MeSH: Medical subject headings
PHC: Primary health care
PFCP: ‘Patient’s Feedback in Clinical Practice’
